# Understanding Potential Exposure of Bicyclists on Roadways to Traffic-Related Air Pollution: Findings from El Paso, Texas, Using Strava Metro Data

**DOI:** 10.3390/ijerph16030371

**Published:** 2019-01-29

**Authors:** Kyuhyun Lee, Ipek N. Sener

**Affiliations:** 1 Texas A&M Transportation Institute, College Station, TX 77843, USA; k-lee@tti.tamu.edu; 2 Texas A&M Transportation Institute, Austin, TX 78752, USA

**Keywords:** bicycle, fitness tracking app, crowdsourced data, Strava, traffic emission, PM_2.5_, health effects, public health, spatial effects

## Abstract

As bicycling on roadways can cause adverse health effects, there is an urgent need to understand how bicycle routes expose bicyclists to traffic emissions. Limited resources for monitoring reveal that bicycle travel patterns may constrain such understanding at the network level. This study examined the potential exposure of bicyclists to traffic-related air pollution in El Paso, Texas, using Strava Metro data that revealed bicycle patterns across the city networks. An initial spatial mapping analysis was conducted to explore the spatial patterns of bicycling and traffic pollutant emission, followed by exploratory descriptive statistics. A spatial bicycle model was then developed to explore factors influencing bicycling activity in El Paso. Analysis results indicated significant associations between greater bicycle volume and both higher levels of particulate matter (PM_2.5_) emissions and more frequent bus services, implying adverse health concerns related to traffic-related air pollution. The results also indicated significant effects of various environmental characteristics (e.g., roadway, bicycle infrastructure, topography, and demographics) on bicycling. The findings encourage extending this study to provide guidance to bicyclists whose regular trips take place on heavily trafficked roads and during rush hours in this region and to evaluate the net health impacts of on-road bicycling for the general population.

## 1. Introduction

### 1.1. Background

Bicycling has numerous benefits that justify efforts to encourage daily bicycling as a desirable transportation and health benefit, but direct exposure to the environment makes bicyclists on the road more vulnerable to traffic-related collisions and emissions. Exposure to traffic emissions is less visible than the risk of traffic collisions, which has probably contributed to the relatively little attention paid to such exposure. While a person is physically active, breathing and ventilation rates increase, and thus the amount of air pollutant inhaled is likely to increase [1]. Even with the expected health benefits of bicycling, poor air quality may generate health trade-offs, such as acute decrements in respiratory function [2,3,4], decreased heart rate variability [5,6], decreased microvascular function [7], and increased risk of myocardial infarction [8]. It has also become widely believed that exposure to traffic-related air pollutants, such as particulate matter with an aerodynamic diameter below 2.5 μm (PM_2.5_), can cause respiratory and chronic diseases [9,10]. Bicyclists who make a daily trip mostly alongside busy routes with heavy traffic and maintain this travel behavior are likely to be at risk of such health problems. However, the levels of exposure to air pollution can vary depending on which routes are taken. Studies comparing different routes revealed that proper route choices play a key role in reducing the levels of exposure to traffic emissions [11,12,13]. Bicycle commuting that takes routes with lower proximity to vehicle traffic could facilitate a reduction in the inhaled particle count in comparison with the counterpart routes of higher proximity [14,15]. Given the risk of exposure to air pollution and possible prevention of the risk, a holistic approach is needed in planning bicycle networks that are accessible, convenient, and safe based on a solid understanding of bicycle travel patterns. 

### 1.2. Influential Factors on Bicycling

Cycling is a human behavior that is a convergence of a myriad of individual (e.g., age, gender, physical abilities, and confidence level), situational (e.g., trip purpose, distance, time, and cost), and environmental (e.g., infrastructure, traffic conditions, and topography) factors [16,17]. Individual sociodemographic features are a major determinant in decisions of which routes to take or whether to ride a bicycle to get to a destination [18,19,20], and psychological factors (such as attitudes and perception) are also important [21,22,23]. Bicyclists generally prefer routes with bicycle infrastructure that can separate and protect them from vehicle traffic [18,20,24,25,26,27,28]. Upslopes, high speeds, and vehicle traffic are often reported as deterrents [18,19,25,26,28,29], but not always [20]. When it comes to situational factors, travel decisions are sensitive to trip time and distance [18,20,30], cost [18,22], and reasons for making the trip [26]. In addition to insights into such factors, an increasing attention to the adverse health effects of exposure to air pollution has encouraged investigating the impacts of air pollution on bicycling decisions. For example, Anowar et al. [30], using stated preference survey data from 695 commuter cyclists, identified how exposure information influences bicyclists’ route choice decisions. While the study results indicated that “travel time and traffic volume remain the most important attributes for commuter cyclists in their route decision”, the authors found that information about air pollution negatively affected route preferences [30]. In an intercept survey of 625 real-world cyclists, 51 percent of survey participants replied that they consider air quality in routing decisions [23]. Stated behavior studies by Cole-Hunter et al. [31] emphasized that bicyclists are willing to avoid air pollution risks during cycling. While stated preferences can capture motivators and deterrents for travel decisions, there may be an information gap between those preferences and real-world travels. Global position system (GPS)-based experiments can minimize the gap by monitoring actual trip patterns, but limited resources hardly permit network-level analysis for the population level [24,26]. Difficulties in acquiring adequate data may be a barrier to studies investigating how revealed bicycle trip patterns are associated with air pollution at the network system level.

### 1.3. Bicycle Data Collection and Strava

Traditionally, transportation planners and practitioners have relied on several monitoring tools to obtain active travel data. The most traditional monitoring method is counting and surveys. Both manual and automated counting are still primarily used to collect active traffic volumes, but they are typically available at a limited number of points and do not provide detailed information (e.g., individual and situational information). By contrast, surveys are preferred when collecting individual and situational information because subjects are asked to describe the information of interest, such as demographic features and experience level. Recently, GPS-based surveys have become more popular because GPS data reveal real-world trip information. Traditional data collection methods are still valuable in monitoring active travelers, but intensive resources, time, and effort frequently limit sample sizes and time/spatial coverage, leading to poor understanding of active travel behavior (for a review of data collection methods for active travelers, see [32]). 

The proliferation of smartphones has created an emerging monitoring method. In their recent study, Lee and Sener [33] conducted a detailed review and evaluation of currently available data collected by various emerging methods and crowdsourced data that take advantage of mobile devices and their current use. As discussed by the authors, GPS-based fitness tracking apps where people upload their physical activities provide new opportunities to obtain revealed travel patterns with relatively less effort than traditional data collection methods. A popular fitness app, Strava, collects users’ mobility information, and its data service, Strava Metro, initially processes the raw data and sells them to data consumers. Because Strava Metro provides spatially and temporally continuous physical activity counts at different geographic units (point, segment, and polygon), these data are useful in capturing interactions between environmental settings and bicyclists across broad regions over a long period. Strava Metro data have been used for different bicycle studies, including, for example, visually depicting cycling patterns [29,34], evaluating the effects of new bike facilities on cycling behaviors [35], analyzing accident risk exposure [36,37], and investigating the associations between environmental characteristics and recreational cycling [38]. 

### 1.4. Strava and Air Pollution

Despite the growing number of application cases, only a few studies have used Strava Metro data to examine ambient pollutant exposure during physical activities. Sun and Mobasheri [39] estimated the instantaneous air pollution exposure of riders using Strava Metro’s node (point) data in Glasgow, Scotland, United Kingdom. The authors calculated the average exposure of commuting and non-commuting cyclists to PM_2.5_ and particulate matter with an aerodynamic diameter below 10 μm (PM_10_) at the node. The outcomes showed that the amount of instantaneous air pollution exposure of commuters was higher than that of non-commuters for both pollutants. A study by Sun et al. [40] assessed the inhaled dose of pollutants for Strava users (bicyclists and pedestrians) in the same area. In this study [40], bicyclists’ average inhaled dose of PM_2.5_ was four times greater than that of pedestrians. The study also located areas that need investment priority (with high-level PM_2.5_ concentration and high-volume cycles/walks). These two studies exemplify the potential of the fitness app data in evaluating the air pollution exposure of active travelers at the area level.

### 1.5. Study Purpose

The current study aimed to contribute to the growing necessity of considering traffic emission exposure in planning bicycle networks. While researchers are interested in how bicyclists are associated with the risk of exposure to traffic-related air pollution, limited resources for data collection are likely to restrict population-level examination across the networks. This study demonstrates how data from Strava can be used to detect potential exposure to traffic air pollution at the street level while bicycling (the prior two studies on air pollution using Strava data are at the area level). This study further examined which environmental characteristics are associated with higher cycling trip volume in El Paso, Texas, using Strava Metro data and is the first trial to look into citywide bicycling patterns in this region. Cities like El Paso, where other data on bicycling are unavailable, may replicate this study since Strava globally collects data anywhere Strava users exist.

## 2. Materials and Methods

### 2.1. Study Area

**Figure 1:**
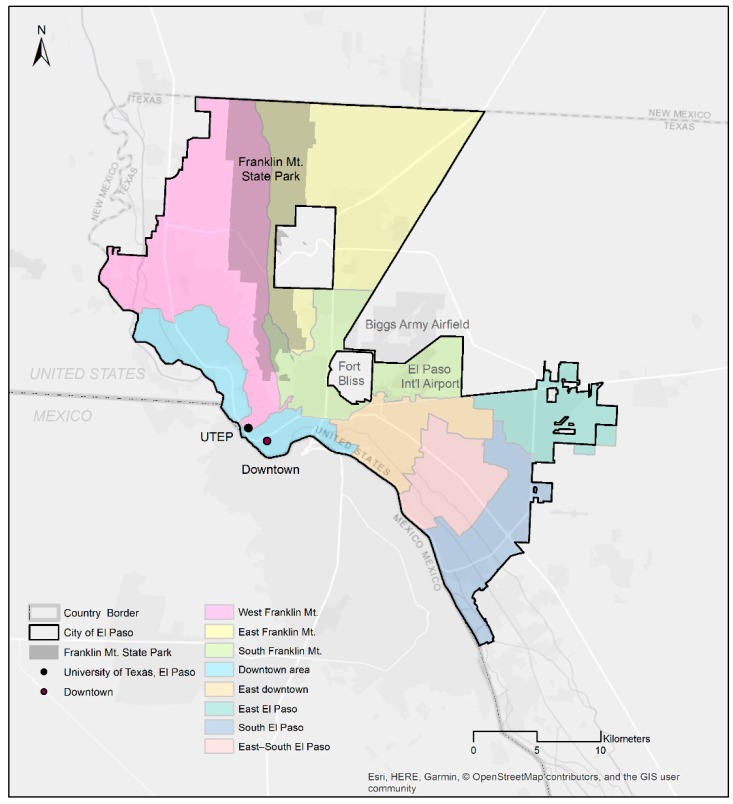
Study area (El Paso, Texas).

The study area was El Paso, the sixth largest city in Texas (by population), situated on the border between the United States and Mexico (Figure 1). This borderland has unique characteristics in geographical, economical, transportation, and public health contexts that need special attention from policy makers. A prominent geographic feature, Franklin Mountains State Park, extends from the northern part of El Paso to the downtown area, nearly dividing the northern part of the El Paso region into the west and east sides. This state park provides the local community with the largest urban park in the El Paso region in which to spend time in nature, doing activities such as bicycling, hiking, and rock climbing. 

Since the enactment of the North American Free Trade Agreement in 1993, El Paso has experienced rapid industrial development, urbanization, economic growth, and surface trade expansion between the United States and Mexico. While providing various opportunities for the region, the significant increase in international border crossings has also yielded several transportation problems, including increased auto dependency and air pollution at the cross-border and surrounding areas. El Paso has failed to meet the National Ambient Air Quality Standards [41], and its annual particle pollution (PM_2.5_) level was ranked eighth worst out of 277 metropolitan areas in the United States [42]. Arid weather, sustained temperature inversions, and occasional sandstorms contribute to deteriorating air quality in the region [43]. 

In conjunction with air pollution issues, sedentary lifestyles and related health problems have required interventions. According to national and regional surveys, El Paso has lower rates of active travel than many other similarly sized urban areas in the United States [44,45]. Over 31 percent of adults in El Paso reported no leisure-time physical activities or exercise in the preceding month in 2015 [46]. Overweight (a body mass index (BMI) between 25 and 29.9) or obese (a BMI above 29.9) adults in the region accounted for about 67 percent of the adult population [47]. These figures highlight the importance of combined tactics to encourage this community to make more active travel choices and to improve environmental conditions.

### 2.2. Data and Variables

#### 2.2.1. Bicycle Count Data

The outcome variable of this study was bicycle counts at street segments for a year. The primary data set, bicycle counts, was obtained from Strava Metro through the Texas Department of Transportation (TxDOT). Strava is a mobile app upon which individuals track their physical activities (e.g., riding, running, walking, and hiking). Bicyclist and pedestrian activities uploaded to the app can be accessed via its data service, Strava Metro. Strava Metro aggregates bicycling and walking records (except records Strava users have made private) to street segments, nodes, and origin and destination polygons, and anonymizes personal information (e.g., which athletes were on the street is de-identified) [48]. This service is commercially available and provides information about physical activity patterns with high temporal and spatial coverage (e.g., wider areas, longer periods, continuous time, and geometry) (https://metro.strava.com). Since “the data provided through Strava Metro has been aggregated and has had individual identifiers removed” [48], the use of this data does not require an institutional review board (IRB) approval. 

Table 1 and Table 2 present statistics of bicycling records and profiles generated by Strava users in the TxDOT El Paso District. From 1 July 2016 to 30 June 2017, 67,824 activities were recorded across the district (on average, one unique user reported about 15 cycling activities). Among the total rides, about 21 percent of trips were made for commuting, and 79 percent were non-commuting trips. The median cycling distance was 32.2 km, and the median time spent riding was 102 min. The majority of the data contributors were male (approximately 81 percent), and most contributors were adults aged 25 to 54 (around 63 percent). According to demographic information from Census data (American Community Survey 2017 (five-year estimates)), 49 percent of residents in El Paso County are male, while 51 percent of all residents are between 25 and 54 [49]. While this comparison shows a bias in Strava users in the El Paso region, the data show similar trip and demographic patterns to other districts in Texas.

Several data processing steps were undertaken to obtain the final analytical sample used for the statistical analysis. In terms of data extraction, street segments within the El Paso city boundary were extracted, and bicycle counts were aggregated for the given year. The street segments delivered by Strava Metro ranged in length from 1 m to 1.5 km (the median was 81 m). To avoid the over-representativeness of very short segments (e.g., 1 m of segments), continuous short segments that had the same features (e.g., speed limit, bus routes, and bike lanes) were merged as one segment (bicycle counts on these segments were averaged). In terms of data cleaning, interstate highways, freeways, and expressways were removed from the sample set for statistical analysis because of the concern about noisy data (e.g., Strava users carrying their phones in their bags or cloth pockets may have forgotten to turn off the app even after reaching their destination, and the app recorded trips in vehicles). In addition, segments matched with traffic emission layers (for PM_2.5_) were only included in the analytical sample set. Since the traffic emission layers were generated based on links that are mostly major thoroughfares, bicycling on trails, most local streets, and no-roads (e.g., parking lots) were excluded in the matching process. The final analytical data included 3501 roadway segments with an average length of 366 m. 

Two caveats are important to note. First, because Strava Metro provided the trip and rider profiles at the district level only, these characteristics were not available to be examined for the final (segment-level) analytical sample. Second, the study was not able to explore the gaps in the general bicycling population or compare the app-based samples with other sources (e.g., automated bicycle counts) due to the lack of adequate bike count data in the El Paso region. As Teri Kaplan, the TxDOT bicycle and pedestrian statewide coordinator, noted in an interview, “There’s actually virtually no real data that’s consistently maintained for bicycles and pedestrians” [50]. On the other hand, when compared with other Strava-based studies, the demographic composition of Strava users in the current study region showed similarity in terms of skewedness toward males and a younger population [29,34,36,38,51].

#### 2.2.2. Explanatory Variables 

Several variables were considered in this study to examine the potential emission exposure and measure the influence of various environmental factors on variations of bicycle volume. Specifically, the variables included roadway characteristics, bicycle infrastructure characteristics, neighborhood demographics, topographical attributes, and emission exposure measures. The variables were selected based on the synthesis of the relevant literature and data availability. Most of the explanatory variables were collected from open sources, such as the City of El Paso, the United States Geological Surveys’ National Elevation Dataset (NED), TxDOT, the U.S. Census Bureau, and the General Transit Feed Specification (GTFS). 

Roadway characteristics included posted speed limits and functional classifications [20,24,25,26,27]. To reflect actual speed limits and eliminate missing values, the posted speed limit information was collected from Google Street View. To the best of our knowledge, there were no major changes in speed limits (that would impact the current analysis) between the year of Google Street View (mostly ranging from 2015 to 2018) and when Strava data were collected (2016–2017). Bicycle infrastructure was classified into two categories: existing or planned. These variables can evaluate not only the effectiveness of current infrastructure but also proposed ones. The values of demographics at the block group level were assigned to the road segments [38]. When no demographic data were available, nearby block group values were applied to avoid generating missing data. As a vertical topographical attribute, maximum elevations and average segment slopes were calculated from the NED digital elevation model at a resolution of 30 m [26,29]. City representative districts where segments belonged served as a horizontal attribute in this analysis. Using the districting map in the current analysis context may seem unusual because this map is not necessarily for bicycle-related policy-making purposes. However, the districting map can be used as an indicator of areas where more or fewer bicyclists make a trip and therefore can be useful for policy decision-making at the district scale (see Figure 1). To examine propensity toward parks, a 61 m buffer from parks was generated [19,44].

To measure potential exposure to traffic emissions, two variables were developed: bus service frequency and PM_2.5_ emission [30]. Bus routes and service schedules were identified from the GTFS and calculated as the total bus service frequency per day. In terms of traffic pollutant, PM_2.5_ was used as a surrogate measure of exposure to near-roadway traffic emissions. While there is no definitive agreement about which pollutant is the best surrogate for traffic air pollution, the main source of PM_2.5_ is vehicle emissions, and PM_2.5_ has been widely used as a proxy for traffic-related air pollution, as discussed earlier [38,39,52,53]. The PM_2.5_ emission data were generated using hourly activity data from the El Paso travel demand model and from the Environmental Protection Agency’s Motor Vehicle Emission Simulator emission factors (2020 analysis year). The hourly PM_2.5_ emissions were then post-processed to be converted to the minute average PM_2.5_ emission per km in gram for 2017. 

All the explanatory variables were attributed to each Strava street segment using ArcGIS software (ArcMap 10.5.1) by ESRI (http://www.esri.com/).

### 2.3. Data Analysis

The data analysis started with a spatial mapping analysis using the raw (unprocessed) data. Three maps were developed to explore the spatial patterns of bicycling and traffic pollutant emissions. The first two maps provided visual indications of variations in total bicycle counts for the given year for commuting and non-commuting trips in the study area. Using the minute average PM_2.5_ (in grams per km) variable, the third map showed where high traffic emission networks were located. The Jenks natural breaks classifier was used to divide data into five classes to cluster ridership and PM_2.5_ emission levels on the maps. 

Next, exploratory descriptive statistics were examined using the final analytical data (*N* = 3501 segments). Finally, using averaged (aggregate) bicycle counts at the segment level, a bicycle demand model was developed to examine the factors influencing bicycle activity (of Strava users) within the study area. Since averaged bicycle counts (for the merged segments) are not (positive) integers and had a non-Gaussian distribution, a least square model was first estimated with log-transformed bicycle counts. Then, a spatial weighting matrix was developed to test spatial autocorrelation statistics (Moran’s I). For spatial correlation analysis, the most typically used matrices are the contiguity matrix and the inverse-distance matrix. To define spatial lags between neighboring roadways that share nodes (ends of a segment), a binary contiguity matrix (W) for first-order neighbors was created (if segments are adjacent, weighting is 1; otherwise 0) [54]. The Moran’s I test result rejected the null hypothesis of the independent residuals, indicating a positive and significant spatial correlation among bicycle counts in street segments. More advanced forms of model specifications were examined, and different variable specifications and alternative functional forms were considered to find the best data fit. Several models were estimated in a stepwise approach by adding the spatial lag of the dependent variable, an autoregressive spatial error, and terms representing the spatial lags of the independent variables using the contiguity matrix W with two generalized spatial stages [55,56,57]. After iterating the model estimation with differently combined explanatory variables, the final variables were selected based on statistical considerations (at the 0.01 level of statistical significance), insights from the literature, intuitiveness, and parsimonious considerations. The modeling efforts were conducted using STATA, Version 15.0 [57].

## 3. Results 

### 3.1. Spatial Patterns of Bicycling and PM_2.5_

**Figure 2:**
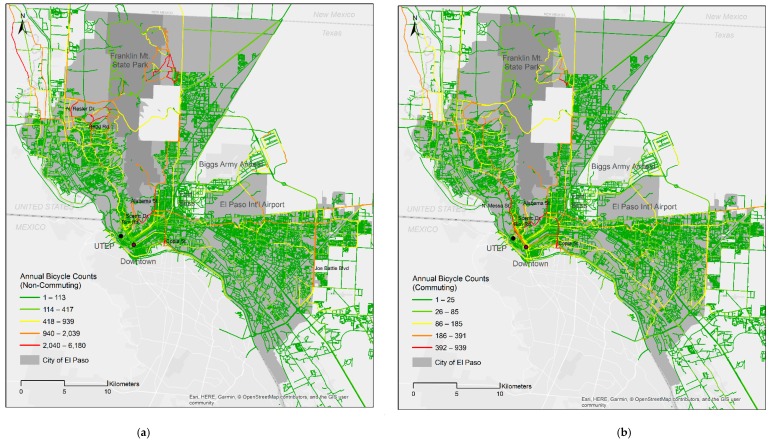
Distribution of bicycle activities by trip purpose (data licensed by Strava (Base map: OpenStreetMap)). (**a**) Annual bicycle counts (non-commuting); (**b**) Annual bicycle counts (commuting).

Figure 2 shows the overall spatial patterns of annual bicycling volumes for non-commuting and commuting trip purposes in El Paso. For the distribution of non-commuting trips (Figure 2a), the most popular routes for Strava bicyclists were trails in the Franklin Mountains State Park (east side of the park). Moving to the west side of the mountains, roadways that provide access to the Franklin Mountains (North Resler Drive and Redd Road) and trails going up the mountain from Redd Road had a high density of bicycle activities for leisure and recreational purposes. The other busiest routes were on the roadways located in the southern end of the mountain range (Rim Road, Scenic Drive, and Alabama Street). Copia Street, which connects to a border-crossing bridge (a port of entry between the United States and Mexico), had a significant volume of cycling activities, implying that many bicyclists use this path to cross the border. The second highest non-commute trip clusters included segments neighboring the busiest roads and arterials (e.g., Joe Battle Boulevard).

Compared to non-commuting trips, commuting patterns showed differences such that commuting routes were more saturated in the center of the city (Figure 2b). While roadways in and around the Franklin Mountains were the most popular routes for non-commuters, ridership for commuting was highly skewed toward the inner city (particularly North Mesa Street toward downtown). The analysis also indicated high bicycling activity at the border crossing, especially for commute purposes. 

**Figure 3:**
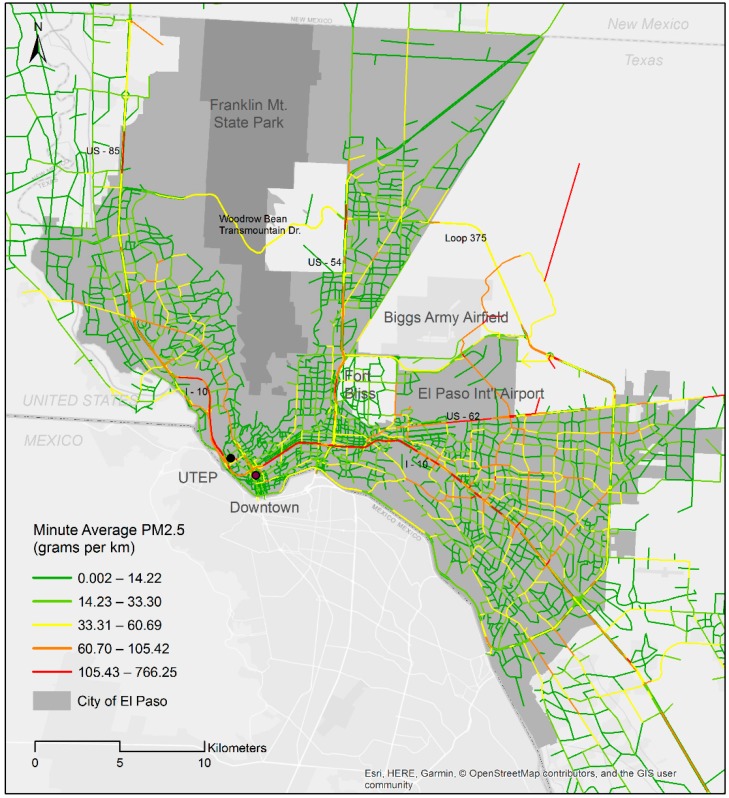
Minute average PM_2.5_ emissions.

Figure 3 illustrates road networks with estimated minute-level PM_2.5_ emission per unit (grams per km) across the El Paso region. Roadways reporting the highest levels of PM_2.5_ are observed among major roads with high vehicle volume (including US Highway 85, Interstate Highway 10, US Highway 54, US Highway 62, and Loop 375). Arterials show the second highest levels of PM_2.5_ emissions. Somewhat surprisingly, roadways passing through the Franklin Mountains (Woodrow Bean Transmountain Drive) are depicted as among the routes with moderate emissions despite low levels of traffic volume.

### 3.2. Descriptive Statistics

Table 3 shows the descriptive statistics for the variables considered in the statistical analysis of this study. As mentioned earlier, the final analytical data used for the statistical analysis included 3501 street segments where, on average, approximately 321 bike trips occurred throughout the year (including both commuting and non-commuting trips). 

Posted speed limits were classified into five categories, as listed in Table 3. The biggest proportion was found in the first speed category (i.e., ≤48 km/h), about 37 percent. As for roadway types based on functional classification, principal arterials accounted for the highest portion of the street segments, about 39 percent. Minor arterials and collectors took up most of the rest, with about 30 percent. 

The data indicated future plans for new bike facilities for more than half of the street segments in the final analytical sample. When it came to existing bicycle infrastructure, roadways were categorized into five types to evaluate the existing facility performance: no bike facility, bike lane, buffered bike lane, off-street path, and shared lane marking. Approximately 19 percent of the street segments were equipped with bike infrastructure. Nearly 9 percent of the street segments had bike lanes, and 5 percent were off-street paths. Buffered bike lanes and shared lane marking constituted about 5 percent when combined. 

In terms of topography, the average elevation was 1183 m, and the mean slope was 1.79 degrees. The highest elevations were observed in the Franklin Mountains and surrounding areas. Around 10 percent of segments were inside or within 61 m of a park, and segments were relatively evenly dispersed across the eight districts (on average, 438 segments per district).

Regarding neighborhood demographics, the percentage of people between age 25 and 54 was, on average, almost 40 percent. Hispanics made up the highest proportion of residents in El Paso—at about 80 percent—followed by Whites and African Americans. The median household income was about $43,400 (in 2015 inflation-adjusted dollars), and on average 24 percent of people were in poverty. Employment density was 699 per square km, and retail job density was 170 per square km.

The mean minute-level PM_2.5_ emissions (per km) were about 34 grams, and on average buses ran 26 times a day.

### 3.3. Bicycle Model

Table 4 presents the results of the final bicycle model at the roadway-segment level. As mentioned earlier, the final specification included variables statistically significant at the 0.01 level except for two variables (that were statistically significant at the 0.1 level of significance) because of their intuitive effects and potential to guide future research efforts in the field. In addition, the assessment of variation inflation factors indicated no collinearity problems. Model estimation considered two types of bicycle activities (non-commuting and commuting), but statistically significant differences in the variables were not noteworthy between the two model results. The final model specification was made for total bicycle trips.

The final model includes three types of spatial terms: spatial lags of dependent variables, spatial lags in error terms, and spatial lags of two explanatory variables (bus frequency and elevation). Adding these spatial lags indicated the best model fit and best controlled the spatial effects of the given data according to the pseudo R-squared and Wald test of spatial terms. The model results showed that sampled cyclists in El Paso preferred certain environmental characteristics and were potentially exposed to traffic emissions. 

For the first category of roadway characteristics, posted speed limits and roadway types were captured as important factors that matter in route choices of Strava users. Strava cyclists preferred roadways with a high speed limit (72 km/h or over) over roadways with a lower speed limit (i.e., 48 km/h or less, 56 km/h, and 64 km/h). The negative signs on the variables of the functional classifications indicate that both principal arterials and minor arterials were preferred over collectors and local streets. 

In terms of bicycle infrastructure characteristics, Strava cyclists favored routes with any type of bike facility over routes with no facilities, with off-street paths having the highest likelihood of use, followed by buffered bike lanes. Such positive and significant associations were found not only for existing infrastructure but also for planned ones. That is, bicycle volumes were significantly higher on the road segments currently having a future plan to install bike facilities. 

For Strava riders in El Paso, vertical topographic characteristics were important factors in predicting their route preferences. Roadways located in higher altitudes had more bicycle trips. In terms of city representative districts, the West Franklin Mountain district had more bicyclists than any other district. This result is quite expected given the potential attractiveness of this area providing access routes to the west side of the mountains, as also indicated by spatial patterns of bicycling in the region. This district also covers the University of Texas-El Paso (UTEP), and the popular cycling routes in the region (see Figure 1 and Figure 2). On the other hand, the East Franklin Mountain district, which is in the northeast part of El Paso, including the east side of the mountains and the desert, was associated with the least amount of bicycling activity in the region. The three districts covering the east downtown, south El Paso, and east-south El Paso parts of the region also had fewer bicycle trips than other districts.

Certain neighborhood demographic characteristics were associated with variations in the number of bicycle trips. The results indicated a positive tendency of bicycling among the block groups, with a higher percentage of the age groups of 35–45 and 45–54 (with the highest positive association among adults aged 45–54). Neighborhoods with higher median household income levels had a greater number of bicycle trips, whereas higher retail job density was associated with fewer bicycle trips. 

Lastly, regarding a central focus of this study, the results of exposure to traffic emission attributes provide valuable insights into the potential adverse health impacts of exposure to traffic-related air pollution while riding along highly trafficked streets. Bicycle trip counts were higher on streets with more frequent bus services. Since buses serve, in general, busy routes with high traffic density, this finding implies that Strava riders take the routes with heavy traffic. The results also indicated that PM_2.5_ emission, one of the primary indicators of air pollution resulting from motorized traffic, was significantly (positively) associated with bicycle activity volumes.

## 4. Discussion

### 4.1. Factors Influencing Bicycle Activity of Strava Users 

The bicycle model findings provide revealed preference toward certain environmental settings, including higher speed limits, principal/minor arterials, steepness, high altitudes, and bicycle infrastructure. Since increasing cycling rates can be an integral component of strategies that generate synergistic effects on multiple social issues in El Paso, the findings should be carefully considered in efforts to promote bicycle activities. 

Preferences to higher speed and arterials (rather than collectors and local streets) may first seem counterintuitive since many other bicycle studies indicate that bicyclists tend to feel uncomfortable with high traffic speeds [25,26,28]. However, considering that the samples were opt-in Strava users who willingly track their physical activities, it can be inferred that the sampled riders are likely to be more experienced/enthusiastic and do not generally escape/avoid high speeds. Rather than feeling safe and comfortable in low-velocity and low-traffic situations, confident riders may be attracted by other variables, such as shorter routes and less pedestrian traffic [21,25]. Such attitudes were also supported in a study that analyzed bicycle route choice preferences in Texas; experienced bicyclists favored relatively higher speed routes over lower speed routes [20]. In addition, Hankey and Lindsey [24] found that principal arterials were associated with increased bicycle traffic volume. In another study by Hankey and colleagues, arterials had more bicycle volume compared to local streets, which might be because this type of road offers travelers efficiency in terms of travel time and distance to their trip destinations [27]. 

Topographic attributes (e.g., steeper slopes and high elevation) were important parameters and are likely associated with characteristics of self-selected Strava riders who tend to physically challenge themselves. While this finding is inconsistent with some previous studies in which sloping segments were found to be deterrents for bicyclists [19,25,26,28,29], it was supported by some other studies and consistent with the sample bias of Strava users (males and younger individuals). For instance, a study by Sener et al. [20] showed that non-commute male riders had a higher inclination for routes with moderate hills over flat terrains and steep hills over moderate hills. Another study that used Strava data [51] also indicated that cyclists may prefer sloping routes due to greater exercise benefits and the potential for rest periods while traveling downhill. Positive correlations with high altitudes can be understood in the same context. The highest landforms in the city are in the Franklin Mountains and adjoining basins, which are major generators of leisure activities (e.g., hiking, biking, and rock climbing) in this region. Strava bicyclists are more likely to be recreational/fitness riders and probably favor the enjoyment of visual and scenic views offered by the mountains and hills. 

As expected from previous studies [18,20,24,25,26,27,28], bike infrastructure had positive effects on increased bicycle volume. Higher bicycle volumes on routes with a proposed bike infrastructure plan support future investment in the proposed plan. When deciding which type of bike facilities should be prioritized, planners and practitioners can use the information about different magnitude of preference by type. The most favored type was off-street paths, followed by buffered bike lanes, shared lane marking, and bike lanes. 

Compared to route characteristics, sociodemographic features in neighborhoods were less determinant factors. Among a rich set of sociodemographic variables, only age, income level, ethnicity, and retail job density were significant. The positive associations with certain age groups (35–45 and 45–54) and Hispanics were in line with the popularity of Strava to people aged 25–54 and the ethnicity composition of the El Paso region. High-income neighborhoods had a greater number of bicycle trips, which is supported by previous findings [24,34] and the fact that health app users tend to have relatively higher income [58]. The negative impact of retail job density was an inverse result from other studies [27,38], but this finding is perhaps again a result of Strava users being recreational/fitness-oriented and seeking networks in more scenic, less crowded, and lower job-activity density areas. 

### 4.2. Potential Exposure to Traffic-Related Air Pollution

The results of positive correlations of bicycle volume with exposure to traffic emission attributes provide insights into the potential adverse health impacts of riding along highly trafficked streets. Buses serve, in general, busy routes with high traffic density. Travel time on such streets is likely to be longer, and frequent stops and departures at bus stops contribute to increased combustion byproducts in the exhaust [11]. As extensively discussed in a Congestion Mitigation and Air Quality Improvement program study [59], PM concentrations can build up, particularly when the bus doors are open and when a line of buses is loading and/or unloading. Therefore, bicycling along busy bus routes raises a concern about increased levels of traffic emission exposure. New technologies or regulatory measures can help reduce vehicle emissions (along these busy bus routes or at bus locations), such as replacing conventional vehicles with alternative fuel vehicles, diesel retrofits, idle reduction, and extreme low-temperature cold-start programs [59]. For effective multimodal transportation systems, transit-bicycle integration is often suggested. A significant relationship between busy routes with frequent bus services and dense bicycle volume implies increased risks of Strava bicyclists in the region being involved in vehicle collisions and inhaling traffic exhaust. The findings highlight the importance of a careful approach to integration of bus networks with bicycling. 

Another worry arises for bicyclists who regularly travel along roadways with heavy traffic in terms of more deleterious effects of accumulated vehicle-related pollution exposure. Previous studies suggested that riding alongside low-traffic routes can significantly reduce air pollution exposure [12,13]. Stated behavior studies indicated bicyclists’ willingness to avoid air pollution risks during cycling. For example, in a questionnaire-based study by Cole-Hunter et al. [31], most of the survey participants indicated a willingness “to adopt risk management strategies (with desired features) if shown to be appropriate and effective.” Thus, bicyclists in the El Paso region need urgent guidance to choose alternative routes if their regular trips take place on heavily trafficked roads and during rush hours. For instance, providing public information about spatial-temporal pollution hot spots and alternative routes to avoid excessive traffic emissions is worth implementing (e.g., [30]). 

In addition to PM_2.5_, five other traffic-related air pollutants were tested (PM_10_, carbon monoxide, nitrogen dioxide, oxides of nitrogen, and volatile organic compounds), and similar results were obtained (every pollutant had a positive/significant association with higher bicycle volume). Although they were not included in the final model estimation due to collinearity issues, the results imply that bicyclists are highly likely to be exposed to a mixture of multiple traffic-emitted pollutants. Intuitively, other vulnerable road users (e.g., pedestrians and runners) are likely to be at the same risk. It is important to continuously monitor and analyze vulnerable road users to create appropriate initiatives for sustainable and healthy transport networks. 

### 4.3. Commuting and Non-Commuting Patterns

The examination of spatial patterns of bicyclists provided additional understanding of bicycling behavior. For example, while Franklin Mountains State Park was the greatest trip generator for non-commute bicycling, roadways in the inner city were popular routes for commuting. Such spatial variations in bicycle ridership by trip purpose are similar to the results of a study of Strava bicycle activities by Sun and Mobasheri [39]. In their study, commute cycling activities were more densely located in the center of the city (in Glasgow) as opposed to non-commute cycling activities. The recognition of the spatial variations by trip type may help develop strategies to improve bicycle environments in a more targeted manner (for instance, if the goal is to promote regular bicycling as part of daily exercise routines, the park may be prioritized for bicycle infrastructure investment). 

Even though spatial patterns showed differences in popular routes between commuters and non-commuters, the modeling approach was not separated by trip type; striking contrasts were not found between the separated estimation results in terms of signs and significance (that might be related to characteristics of Strava data and users of the Strava app). Though not reported here, the commuting counts model showed a larger magnitude of the coefficient on bus frequency and PM_2.5_ emission variables than the non-commuting count model did. As Sun and Mobasheri [39] suggested, bicyclists are likely to be differently exposed to air pollution by trip purpose. Different levels of being exposed to traffic-related air pollution will need to be considered in order to implement more effective strategies.

### 4.4. Limitations and Future Work 

Although providing valuable insights, the results gained from Strava bicyclists may not be generalized to the general bicycle population because of the inherent sample bias of Strava athletes (e.g., recreational riders rather than utilitarian riders and males rather than females). Developing robust data fusion techniques will help supplement the bias issue and remove the uncertainty in representing the total population. In spite of the limitations, Strava data can significantly improve research capabilities for transportation and public health agencies having limited data. For instance, examining Strava bicycle footprints can be a preliminary step in identifying bicycle patterns throughout a city or county and for setting a counting system, especially for cities like El Paso that have few options for data collection. 

Analysis results indicate the necessity for further study of public health implications. Although air pollution may negate the health benefits from bicycling, studies argue that health and societal gains greatly outweigh the risks [60,61], and physical activity may alleviate the detrimental impact of traffic-related air pollution on respiratory health [4,6]. Given that inhaled doses of pollutants are the product of ventilation rate, duration of exposure, and pollutant concentration [61] and that other factors can influence the level of exposure of bicyclists to traffic-related air pollution (e.g., individual health conditions, proximity to vehicle emissions, wearing mask or helmet when riding), it will also be valuable to extend this study with a focus on evaluating the net health impacts of bicycling. Another objective of future research can be drawn from the context of the border region between the United States and Mexico. Future work may consider cross-border travel patterns with a focus on non-motorized modes of travel (walking and biking) using Strava data. How the patterns change in relation to relevant policy efforts (e.g., developing a multimodal transportation system to reduce automobile dependency) across time can be of future interest since Strava data are being updated every day. Finally, future research may delve into trade-offs between air pollution exposure and other important attributes (e.g., travel time and distance) in route decisions (and by different trip purposes). Evaluating “value of clean ride” [30]—in other words, whether and to what extent cyclists are willing to take routes with less traffic-generated pollution—could provide valuable and practical insights into route choices. 

## 5. Conclusions

Despite the health benefits of exercise, bicycling in a polluted atmospheric environment can cause adverse health impacts. Understanding how people make bicycle trips and how much they are exposed to the less visible threats of traffic is a critical part of decision-making, especially for cities faced with public health and environmental issues like the El Paso region. However, with limited data resources on bicycle users, such efforts might be constrained. This study examined the potential exposure of bicyclists to traffic-emitted pollutants and the influential factors affecting link-level bicycle volume in El Paso using fitness tracking app data. The findings of significant associations of bicycle volume with higher levels of PM_2.5_ emissions and more frequent bus services imply the possible health trade-offs of bicycling in this region. Transportation and public health authorities in the El Paso region should consider guiding bicyclists to alternative routes if their regular trips take place on heavily trafficked roads or during rush hours. The study results can also be a basis of future work for policy makers to design safer, healthier, and better adopted bicycle networks. An evaluation of traffic-related air pollution, integrated with a knowledge of influential factors on bicycle trip-making decisions and patterns, will play a key role in the development and calibration of regional network models.

## Figures and Tables

**Table 1:** Bicycle trip summary within the TxDOT El Paso District.

Category	Summary
Rider Ids ^1^	4459
Activities ^2^	67,824 (100.0%)
Commute	14,259 (21.0%)
Non-commute	53,565 (79.0%)
Average distance	32.2 km
Median distance	26.0 km
Average time	118 min (16.4 km/h)
Median time	102 min (15.4 km/h)

^1^ The number of unique user IDs that had a ride start in the El Paso District. ^2^ The number of unique bicycling activity IDs that had a ride start in the El Paso District.

**Table 2:** Bicycle rider summary within the TxDOT El Paso District.

Age	Male	Female	Total
<25	202 (6.0%)	47 (6.1%)	249 (6.0%)
25–34	663 (19.7%)	161 (20.7%)	824 (19.9%)
35–44	797 (23.7%)	184 (23.7%)	981 (23.7%)
45–54	659 (19.6%)	142 (18.3%)	801 (19.3%)
55–64	258 (7.7%)	44 (5.7%)	302 (7.3%)
65+	65 (1.9%)	8 (1.0%)	73 (1.8%)
Blank age	721 (21.4%)	190 (24.5%)	911 (22.0%)
Total	3365 (81.3%)	776 (18.7%)	4141 (100.0%) ^1^

^1^ Blank gender (318) is not included.

**Table 3:** Descriptive statistics of key variables.

Category	Variable Descriptions		Continuous				Categorical
			Mean	STD	Min.	Max.	Obs. (%)
Bicycle count	Annual bicycle counts for analytic sample (*N* = 3501 street segments)		321.42	452.24	1	4371	—
Roadway characteristics	Posted speed limit	48 km/h (30 mph) or less	—	—	—	—	1293 (36.9%)
		56 km/h (35 mph)	—	—	—	—	592 (17.0%)
		64 km/h (40 mph)	—	—	—	—	731 (20.9%)
		72 km/h (45 mph)	—	—	—	—	646 (18.5%)
		80 km/h (50 mph or over)	—	—	—	—	237 (6.8%)
	Roadway type	Principal arterial	—	—	—	—	1358 (38.8%)
		Minor arterial	—	—	—	—	993 (28.4%)
		Collector	—	—	—	—	1089 (31.1%)
		Local street	—	—	—	—	61 (1.7%)
	Segment length (m)		366.51	327.11	31.15	3661.98	—
Bicycle infrastructure characteristics	Planned bike facilities		—	—	—	—	1954 (55.8%)
	Existing bike facilities	No bike facility	—	—	—	—	2837 (81.0%)
		Bike lane	—	—	—	—	309 (8.8%)
		Shared lane marking	—	—	—	—	52 (1.5%)
		Buffered bike lane	—	—	—	—	128 (3.7%)
		Off-street path	—	—	—	—	175 (5.0%)
Topographical attributes	Elevation (m)		1183.49	48.11	1117.06	1599.75	—
	Segment slope (%)		1.79	2.03	0.10	30.43	—
	Park within 61 m (200 ft)		—	—	—	—	385 (10.0%)
	District	West Franklin Mt.	—	—	—	—	369 (10.5%)
		East Franklin Mt.	—	—	—	—	371 (10.6%)
		South Franklin Mt.	—	—	—	—	529 (15.1%)
		Downtown area	—	—	—	—	817 (23.3%)
		East downtown	—	—	—	—	452 (12.9%)
		East El Paso	—	—	—	—	225 (6.4%)
		South El Paso	—	—	—	—	319 (9.1%)
		East-South El Paso	—	—	—	—	419 (12.0%)
Neighborhood demographics	Age/gender	People aged 25–34 (%)	0.14	0.06	0.01	0.41	—
		People aged 35–44 (%)	0.12	0.05	0	0.27	—
		People aged 45–54 (%)	0.12	0.05	0	0.34	—
		Male (%)	0.49	0.08	0.26	0.84	—
	Race/ethnicity	Hispanic (%)	0.80	0.17	0.16	1	—
		White (%)	0.14	0.12	0	0.65	—
		African American (%)	0.03	0.05	0	0.35	—
		Asian (%)	0.01	0.03	0	0.19	—
	Socio-economics	Median household income ($10,000)	4.34	2.30	0.95	14.29	—
		Below poverty level (%)	0.24	0.19	0	0.81	—
		College graduate/above (%)	0.51	0.20	0.02	0.95	—
		Single unit housing (%)	0.60	0.30	0	1	
		Employment density (per sq. km)	669.39	468.27	4.95	3146.70	—
		Retail job density (per sq. km)	169.61	352.76	0	3543.27	—
Emission exposure measures	Bus frequency (times per day)		26.24	47.58	0	873	—
	Bus routes (count)		1.21	1.73	0	22	—
	PM _2.5_ (gram/min/km)		33.52	48.18	2.3 × 10^−3^	766.25	—

**Table 4:** Bicycle model results.

Category	Variable Name		Estimate	T-Stat.
Roadway characteristics	Posted speed limit	48 km/h (30 mph) or less	−0.327	−5.56
		56 km/h (35 mph)	−0.481	−7.22
		64 km/h (40 mph)	−0.298	−4.84
	Roadway type	Collector	−0.569	−10.39
		Local street	−1.361	−8.05
Bicycle infrastructure characteristics	Planned bike facilities		0.678	12.44
	Existing bike facilities	Off-street path	1.474	13.54
		Buffered bike lane	1.340	11.16
		Shared lane marking	1.047	6.10
		Bike lane	0.886	10.19
Topographical attributes	Elevation (100 m)		0.402	6.77
	Segment slope (%)		0.017	1.85
	District	West Franklin Mt.	0.424	4.93
		East Franklin Mt.	−0.796	−8.84
		East downtown	−0.492	−6.59
		South El Paso	−0.585	−6.67
		East-South El Paso	−0.697	−8.50
Neighborhood demographics	Age	People aged 35–44 (%)	0.702	1.75
		People aged 45–54 (%)	2.030	4.23
	Ethnicity	Hispanic (%)	0.465	2.72
	Socioeconomics	Median household income ($10,000)	0.096	7.06
		Retail job density (1000 per sq. km)	−0.259	−3.94
Emission exposure measures	Bus frequency (100 times per day)		0.339	7.32
	(Natural log of) PM_2.5_ (gram/min/km)		0.146	9.11
Constant			−1.496	−2.13
Spatial terms		Spatial error	0.900	12.06
		Spatial lag parameter of bike counts	0.381	4.62
		Spatial lag parameter of bus frequency	−0.532	−3.41
		Spatial lag parameter of elevation	−0.123	−3.60

Notes: (1) Sample size is 3501; (2) Pseudo R-sq. is 0.40; (3) Chi-sq. statistic for Moran’s I is 1107.66 (*p*-value = 0.0000); (4) Chi-sq. statistic for Wald test of spatial terms is 747.52 (*p*-value = 0.0000).
